# Pattern of bacterial and fungal pathogen in patients with high risk for invasive fungal disease in an indonesian tertiary care hospital: an observational study

**DOI:** 10.11604/pamj.2018.29.60.11931

**Published:** 2018-01-22

**Authors:** Gurmeet Singh, Stephanie Gita Wulansari

**Affiliations:** 1 Respirology and Critical Illness Division, Internal Medicine Department, Faculty of Medicine Universitas Indonesia/Cipto Mangunkusumo Hospital, Jakarta, Indonesia

**Keywords:** Candida tropicalis, gram-negative bacteria, invasive fungal disease, Klebsiella pneumoniae, pattern of pathogen

## Abstract

**Introduction:**

In critically ill patients, there is a defect in host defense mechanism resulting in increased susceptibility to bacterial and fungal infection. The pattern of organisms causing infections varies between different countries and hospitals; therefore it is important that every hospital generates antibiograms to guide healthcare professionals during treatment with optimal choice of antibiotics. Our study aimed to described the pattern of fungal and bacterial pathogen in patients with high risk for invasive fungal disease (IFD).

**Methods:**

An observational study was conducted in Cipto Mangunkusumo Hospital, Jakarta, Indonesia, within March-September 2015. Specimens were taken from blood, sputum, endotracheal aspiration, bronchoalveolar lavage (BAL), urine, pus and drainage fluid/surgical tissue specimen on 5^th^-7^th^ day of hospitalization. Samples were cultured onto suitable culture media and bacterial isolates were identified using standard biochemical methods.

**Results:**

Bacteria and Candida sp. were isolated from 153 patients. C. tropicalis (44.31%) was the commonest fungal isolated. Incidence of gram-negative bacteria was higher than gram-positive bacteria. Klebsiella pneumonia was the most common gram-negative bacteria isolated, where as Enterococcus faecalis for gram-positive bacteria.

**Conclusion:**

Critically ill patients were vulnerable to contracted fungal and bacterial pathogen. Candida non-albicans and Gram-negative bacteria were the most common pathogen detected among critically ill patients with high risk for IFD.

## Introduction

Invasive fungal disease (IFD) is a major cause of morbidity and mortality in the critically ill and imunocompromised patients, most commonly caused by *Candida sp* [1-4]. To improve patient prognosis, early diagnosis of IFD is needed to start early antifungal therapy. Leon et al created a scoring system called *Candida score* to identify patients with high risk of IFD, thus patients can be given early antifungal therapy if required. Patients with a *Candida score* >2.5 are of high risk of contracting IFD [5, 6]. Critically ill patients are susceptible not only to fungal infection but also bacterial and opportunistic infections. In critically ill patients, there is a defect in the host defense mechanisms due to immune-suppressive effects of the underlying diseases, recent surgery, trauma and concurrent drug therapy, which results in increased susceptibility to infections. Opportunistic infection also occurs due to exposure to various invasive devices. Furthermore, prior colonization is an important predisposing factor for nosocomial infections in ICU [1, 7]. The pattern of organisms causing infections varies between different countries and hospitals; therefore it is important that every hospital generates antibiograms to guide healthcare professionals during treatment with optimal choice of antibiotics. In Europe and US, epidemiological data of pathogens were updated periodically by health agency like Center for Disease Control (CDC) [8]. Data regarding fungal and bacterial pathogens in Indonesia are still scarce. The main objective of this study is to determine the pathogen profile in critically ill patients with high risk of invasive fungal disease in an Indonesian tertiary care hospital. Better understanding of pathological pattern can help determine appropriate antibiotic and antifungal administration especially in critically ill patients in order to decrease the morbidity and mortality.

## Methods

Study was conducted in ICU/HCU and common ward of Dr. Cipto Mangunkusumo Hospital, a tertiary care hospital in Jakarta, Indonesia. Samples of patients admitted during March-September 2015 were included in present study. A total of 218 critically ill patients aged ≥ 18 years with high IFD risk factor were taken as a sample. *Candida score* by Leon [5] was used to determine patients with high IFD risk factor. Patients who had total parenteral nutrition, surgery and multifocal *Candida* colonization each gained 1 score. Patients who developed severe sepsis gained 2 score. A total score of ≥ 3 confirmed patients with invasive candidiasis who will benefit from early antifungal treatment, although still highly improbable in patients with *Candida score* < 3 [5, 6]. Exclusion criteria were patient/family who refused to take part in the research, passed away or discharged before sampling on day 5-7 of treatment (drop out), incomplete medical record and patient with no diagnosis of infection. Bacterial and fungal infections were studied in detail. Samples were taken from blood (max. 20ml), respiratory specimen (sputum, endotracheal aspiration, bronchoalveolar lavage [BAL]), urine (50 ml), pus and drainage fluid/surgical tissue specimen on treatment day 5-7. Sampling technique was done according to the operational standards of prevention and control of Nosocomial Infection. Blood and body fluid specimen were collected under aseptic conditions in the BACTEC culture vial and processed in microbiology division, clinical pathology department. Bronchoalveolar lavage specimens were processed in Parasitology Department. Cytology and histopathology specimens were processed in pathology anatomy department. Samples were taken using consecutive sampling. Primary data which has been obtained was processed using Microsoft Excel 2007. Statistical analysis used is the numerical data presented as median with standard deviation. This research gained ethical approval (No 182/UN2.F1/ETIK/2015) from Ethical Research Committee Universitas Indonesia. All data were kept confidential by the researchers.

## Results

**Figure 1:**
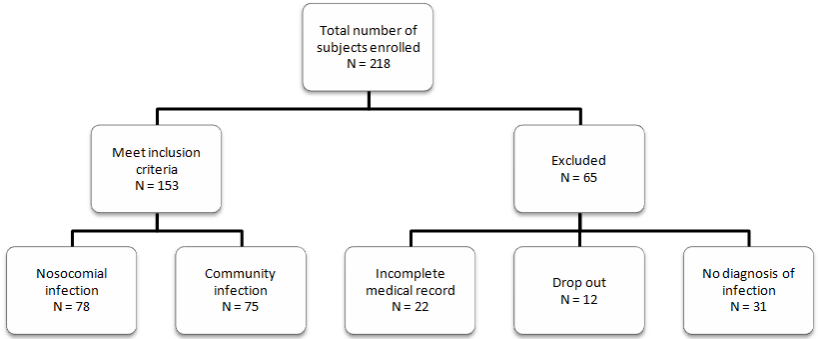
Study design

Two hundred and eighteen subjects were enrolled in this study, 153 subjects fulfilled the inclusion criteria as seen in Figure 1. Majority were male (59.48%) with median age 57 (range, 16-96) year. Median Leon score was 2 (range score, 2-5). Length of hospital stay was 19 (range, 2-84) days. Critically ill high risk IFD patients had a higher risk of nosocomial infection (50.98%). Mortality rate was higher in nosocomial infection with 50.98%. Patient's characteristics are summarized in Table 1. There were 259 specimens collected from 153 patients. From 259 specimens, 139 were collected from 78 patients with nosocomial infection and 120 from 75 patients with community infection. Those specimens divided into three ethiology: medical, surgical and trauma. From 259 specimens, 43 were infected with gram-positive bacteria, 128 with gram-negative bacteria and 88 with fungal pathogen. We found *Enterococcus faecalis* (N = 7, 16.28%), *Klebsiella pneumoniae* (N = 33, 25.78%) and *Candida tropicalis* (N = 39, 44.31%) as the most common gram-positive, gram-negative and fungal pathogen found, respectively. The data of all pathogens found in critically ill patients are described in Table 2. In nosocomial infections, the most common pathogen found based on medical ethiology were *Bacillus sp* and *Enterococcus faecalis* for gram-positive bacteria, *Klebsiella pneumoniae* for gram-negative bacteria and *Candida albicans* for fungal pathogen. For trauma, there were only 4 specimens collected and the numbers of pathogen found were equally divided between 4 kinds of gram-negative bacteria pathogen found. For surgical, the most common pathogen found were *Enterococcus faecium* for gram-positive bacteria, *Klebsiella pneumonia*for gram-negative bacteria and *Candida parapsilosis* for fungal pathogen. The pattern of pathogen found in nosocomial patients are described in Table 3, Table 3 (suite). In community infections, the most common pathogen found in medical ethiology were *Enterococcus faecalis*, *Staphylococcus aureus* and *Staphylococcus epidermidis* for gram-positive bacteria; *Klebsiella pneumoniae* and *Pseudomonas aeruginosa* for gram-negative bacteria; and *Candida tropicalis* for fungal pathogen. For trauma, *Klebsiella pneumoniae* was the only pathogen found. For surgical, no dominant gram-positive bacteria pathogen were found, although *Klebsiella pneumoniae*and *Candida tropicalis* were the most common gram-negative bacterial and fungal pathogen found, respectively. The pattern of pathogen found in community infection patients are described in Table 4, Table 4 (suite).

**Table 1:** Patient characteristics

Patient characteristics	Result
**Gender, N (%)**	
Male	91 (59.48)
Female	62 (40.52)
Age (year), median (min-max)	57 (16-96)
Leon score, median (min-max)	2 (2-5)
Length of stay (days), median (min-max)	19 (2-84)
**Type of infection, N (%)**	
Community	75 (49.02)
Medical	56 (74.67)
Surgical	18 (24)
Trauma	1 (1.33)
Nosocomial	78 (50.98)
Medical	58 (74.36)
Surgical	4 (5.13)
Trauma	16 (20.51)
**Mortality, N (%)**	
Community	75 (49.02)
Survive	41 (54.67)
Died	34 (45.33)
Nosocomial	78 (50.98)
Survive	40 (51.28)
Died	38 (48.72)

**Table 2:** Distribution of organisms isolated from 153 critically ill patients with high risk IFD

Gram-Positive Bacteria		Gram-Negative Bacteria	
*Bacillus sp*	5 (11.63)	*Acinetobacter baumannii*	2 (1.56)
***Enterococcus faecalis***	7 (16.28)	*Acinetobacter baumannii (anitratus)*	16 (12.50)
*Enterococcus faecium*	6 (13.95)	*Acinetobacter lwoffii*	7 (5.47)
*Enterococcus sp*	3 (6.98)	*Acinetobacter sp*	6 (4.69)
*Staphylococcus aureus*	3 (6.98)	*Citrobacter diversus*	1 (0.78)
*Staphylococcus aureus (MRSA)*	3 (6.98)	*Citrobacter freundii*	2 (1.56)
*Staphylococcus epidermidis*	6 (13.95)	*Enterobacter*	1 (0.78)
*Staphylococcus epidermidis (MRSE)*	5 (11.63)	*Enterobacter aerogenes*	9 (7.03)
*Staphylococcus saphrophyticus*	1 (2.32)	*Enterobacter cloacae*	1 (0.79)
*Staphylococcus saphrophyticus (MRSS)*	1 (2.32)	*Escherichia coli*	12 (9.38)
*Streptococcus alfahemolyticus*	3 (6.98)	*Klebsiella oxytoca*	1 (0.78)
Total Gram-Positive Bacteria	43 (100)	*Klebsiella pneumoniae*	33 (25.78)
**Fungal**		*Klebsiella sp*	7 (5.47)
*Candida albicans*	35 (39.77)	*Proteus mirabilis*	3 (2.34)
***Candida tropicalis***	39 (44.31)	*Pseudomonas aeruginosa*	22 (17.19)
*Candida parapsilosis*	4 (4.55)	*Pseudomonas sp*	3 (2.34)
*Candida crusei*	5 (5.68)	*Salmonella sp*	1 (0.78)
*Candida glabrata*	1 (1.14)	*Serratia liquefaciens*	1 (0.78)
*Undifferentiated Candida sp.*	4 (4.55)		
Total Fungal Pathogen	88 (100)	Total Gram-Negative Bacteria	128 (100)

**Table 3:** Pattern of nosocomial bacterial and fungal pathogens

	Organism	Blood	Endotracheal Aspiration	BAL	Sputum	Urine	Pus	Surgical Drain	Total
**Medical**									
**Gram + Bacteria**	*Bacillus sp*	2	-	-	-	2	-	-	4
	*Enterococcus faecalis*	-	-	2	-	2	-	-	4
	*Enterococcus faecium*	-	-	-	-	3	-	-	3
	*Enterococcus sp*	1	-	-	-	1	-	-	2
	*Staphylococcus aureus (MRSA)*	2	-	-	-	-	-	-	2
	*Staphylococcus epidermidis*	1	-	1	1	-	-	-	3
	*Staphylococcus epidermidis (MRSE)*	1	-	-	-	1	-	-	2
	*Staphylococcus saphrophyticus (MRSS)*	-	-	-	1	-	-	-	1
	*Streptococcus alfahemolyticus*	-	-	1	-	-	-	-	1
	***Sub Total***	7	-	4	2	9	-	-	22 (22)
**Gram - Bacteria**	*Acinetobacter baumannii (anitratus)*	2	-	1	1	-	-	-	4
	*Acinetobacter lwoffii*	-	-	2	-	1	-	-	3
	*Acinetobacter sp*	1	-	1	1	-	-	-	3
	*Enterobacter aerogenes*	-	-	1	1	-	-	-	2
	*Enterobacter cloacae*	-	-	-	1	-	-	-	1
	*Escherichia coli*	1	-	-	1	-	-	-	2
	*Klebsiella pneumoniae*	1	-	3	7	2	-	-	13
	*Klebsiella sp*	-	-	1	1	-	-	-	2
	*Pseudomonas aeruginosa*	-	-	1	7	-	-	-	8
	*Pseudomonas sp*	-	-	-	1	1	-	-	2
	***Sub Total***	5	-	10	21	4	-	-	40 (40)
**Fungal**	C. albicans	-	-	5	2	10	-	-	17
	C. tropicalis	2	-	5	3	2	-	-	12
	C. crusei	1	-	-	1	3	-	-	5
	Undifferentiated Candida sp.	1	-	-	-	3	-	-	4
	***Sub Total***	4	-	10	6	18	-	-	38 (38)
***All Isolates, N(%)***		16 (16)	-	24 (24)	29 (29)	31 (31)	-	-	100 (100)

**Table 3 (suite):** Pattern of nosocomial bacterial and fungal pathogens

Trauma									
**Gram - Bacteria**	*Acinetobacter baumannii*	-	-	-	1	-	-	-	1
	*Acinetobacter baumannii (anitratus)*	-	-	1	-	-	-	-	1
	*Acinetobacter sp*	1	-	-	-	-	-	-	1
	*Pseudomonas aeruginosa*	-	-	-	-	1	-	-	1
***All Isolates, N(%)***		1 (25)	-	1 (25)	1 (25)	1 (25)	-	-	4 (100)
**Surgical**									
**Gram + Bacteria**	*Bacillus sp*	-	-	-	1	-	-	-	1
	*Enterococcus faecium*	-	-	-	-	1	1	-	2
	*Staphylococcus aureus*	1	-	-	-	-	-	-	1
	*Staphylococcus epidermidis (MRSE)*	1	-	-	-	-	-	-	1
	*Streptococcus alfahemolyticus*	-	-	-	-	1	-	-	1
	***Sub Total***	2	-	-	1	2	1	-	6 (17.1)
**Gram - Bacteria**	*Acinetobacter baumannii (anitratus)*	-	-	1	1	2	-	-	4
	*Acinetobacter lwoffii*	-	-	1	1	-	-	-	2
	*Acinetobacter sp*	-	-	1	-	-	-	-	1
	*Enterobacter aerogenes*	-	-	-	3	-	1	-	4
	*Escherichia coli*	-	-	1	-	-	-	-	1
	*Klebsiella pneumoniae*	-	-	-	4	-	1	-	5
	*Pseudomonas aeruginosa*	-	-	-	4	-	-	-	4
	*Serratia liquefaciens*	-	-	1	-	-	-	-	1
	***Sub Total***	-	-	5	13	2	2	-	22 (62.9)
**Fungal**	C. albicans	-	-	1	-	1	-	-	2
	C. tropicalis	-	-	1	1	-	-	-	2
	C. parapsilosis	3	-	-	-	-	-	-	3
	***Sub Total***	3	-	2	1	1	-	-	7 (20)
***All Isolates, N(%)***		5 (14.3)	-	7 (20)	15 (42.9)	5 (14.3)	3 (8.5)	-	35 (100)
***Total Specimens, N(%)***		22 (15.8)	-	32 (23)	45 (32.4)	37 (26.6)	3 (2.2)	-	139 (100)

**Table 4:** Pattern of community bacterial and fungal pathogens

	Organism	Blood	Endotracheal Aspiration	BAL	Sputum	Urine	Pus	Surgical Drain	Total
**Medical**									
**Gram + Bacteria**	*Enterococcus faecalis*	-	-	-	-	2	-	-	2
	*Enterococcus faecium*	-	-	-	-	1	-	-	1
	*Enterococcus sp*	-	-	-	-	1	-	-	1
	*Staphylococcus aureus*	1	-	-	-	-	1	-	2
	*Staphylococcus epidermidis*	-	-	2	-	-	-	-	2
	*Staphylococcus epidermidis (MRSE)*	-	-	-	-	1	-	-	1
	*Streptococcus alfahemolyticus*	-	-	-	-	1	-	-	1
	***Sub Total***	1	-	2	-	6	1	-	10 (12.3)
**Gram - Bacteria**	*Acinetobacter baumannii (anitratus)*	-	-	1	2	-	-	-	3
	*Acinetobacter lwoffii*	1	-	-	-	-	-	-	1
	*Acinetobacter sp*	-	-	-	1	-	-	-	1
	*Enterobacter*	-	-	-	-	1	-	-	1
	*Enterobacter aerogenes*	-	-	2	-	-	-	-	2
	*Escherichia coli*	1	-	-	2	2	1	1	7
	*Klebsiella oxytoca*	-	-	1	-	-	-	-	1
	*Klebsiella pneumoniae*	-	-	3	4	1	-	-	8
	*Klebsiella sp*	-	-	1	-	-	-	1	2
	*Proteus mirabilis*	-	-	-	-	1	-	-	1
	*Pseudomonas aeruginosa*	-	-	3	5	-	-	-	8
	*Pseudomonas sp*	-	-	-	-	1	-	-	1
	*Salmonella sp*	1	-	-	-	-	-	-	1
	***Sub Total***	3	-	11	14	6	1	2	37 (45.7)
**Fungal**	C. albicans	-	-	5	2	7	-	-	14
	C. tropicalis	-	-	4	3	12	-	-	19
	C. glabrata	-	-	1	-	-	-	-	1
	***Sub Total***	-	-	10	5	19	-	-	34 (42)
***All Isolates, N(%)***		4 (5)	-	23 (28.4)	19 (23.4)	31 (38.2)	2 (2.5)	2 (2.5)	81 (100)

**Table 4 (suite):** Pattern of community bacterial and fungal pathogens

Trauma									
	*Klebsiella pneumoniae*	1	-	1	-	-	-	-	2
***All Isolates, N(%)***		1 (50)	-	1 (50)	-	-	-	-	2 (100)
**Surgical**									
**Gram + Bacteria**	*Enterococcus faecalis*	1	-	-	-	-	-	-	1
	*Staphylococcus aureus (MRSA)*	-	-	-	-	-	1	-	1
	*Staphylococcus epidermidis*	-	-	-	-	1	-	-	1
	*Staphylococcus epidermidis (MRSE)*	-	-	-	1	-	-	-	1
	*Staphylococcus saphrophyticus*	-	-	-	-	1	-	-	1
	***Sub Total***	1	-	-	1	2	1	-	5 (13.5)
**Gram - Bacteria**	*Acinetobacter baumannii*	-	-	1	-	-	-	-	1
	*Acinetobacter baumannii (anitratus)*	1	1	1	1	-	-	-	4
	*Acinetobacter lwoffii*	-	-	-	-	-	1	-	1
	*Citrobacter diversus*	-	-	-	-	-	1	-	1
	*Citrobacter freundii*	-	-	-	-	-	2	-	2
	*Enterobacter aerogenes*	-	-	-	-	1	-	-	1
	*Escherichia coli*	-	-	-	-	1	-	-	1
	*Klebsiella pneumoniae*	-	-	3	2	-	-	-	5
	*Klebsiella sp*	-	1	-	-	-	2	-	3
	*Proteus mirabilis*	-	-	1	1	-	-	-	2
	*Pseudomonas aeruginosa*	-	-	-	1	-	-	-	1
	***Sub Total***	1	2	6	7	-	7	-	23 (62.2)
**Fungal**	C. albicans	-	-	2	-	-	-	-	2
	C. tropicalis	-	1	1	-	3	1	-	6
	C. parapsilosis	-	-	-	-	1	-	-	1
	***Sub Total***	-	1	3	-	4	1	-	9 (24.3)
***All Isolates, N(%)***		2 (5.4)	3 (8.1)	9 (24.3)	8 (21.7)	6 (16.2)	9 (24.3)	-	37 (100)
***Total Specimens, N(%)***		7 (5.8)	3 (2.5)	33 (27.5)	27 (22.5)	37 (30.8)	11 (9.2)	2 (1.7)	120 (100)

## Discussion

In this study, we describe the bacterial and fungal pathogens found in critically ill patients with high risk for invasive fungal disease. We found that *Enterococcus faecalis*, *Klebsiella pneumoniae*, and *Candida tropicalis* as the most common gram-positive, gram-negative and fungal pathogen found, respectively. This result was acquired by comparing all specimens without excluding nosocomial and community infection. In addition, we found that there was higher rate of gram-negative bacterial infection. Overall, incidence of *Candida non-albicans*as a causal fungal pathogen overrides *Candida albicans*. This is similar with a study by Slavin MA [9] and Chakrabarti A [9]. A study by Tritipwanit et al [10] also reported a significant increase of Candida infection in Thailand. They observed episodes of Candidemia every year and reported an increase from 9 patients during 1991-1992 to 72 patients during 2001-2003 in King Chulalongkorn Memorial Hospital. In addition, they reported a shift of dominant Candidemia pathogens, from *Candida albicans* towards *Candida non-albicans* since 2001. They found that *C. tropicalis*, *C. parapsilosis* and *C. guilliermondii* were the most common *Candida non-albicans*pathogen [10]. In comparison, Siriraj Hospital in Thailand reported *C. tropicalis*, *C. parapsilosis* and *C. glabrata*as the most common *Candida-non albicans* pathogen found; and Ramathibodi Hospital reported *C. parapsilosis*, *C. tropicalis* and *C. glabrata* [10]. This finding may be attributed to the similarity in geographical and economic condition of both countries. A study in North America also reported an increase in *Candida non-albicans*infections [11]. They mentioned the decreasing number of *C. albicans* isolates from 64% in Canada and 48.9% in USA in the 1990s, to 38% in 2008-2011, in comparison with the increasing ratio of *C. tropicalis*, *C. parapsilosis* and *C. glabrata* [11] On the contrary, a study from Latin America still reported *C. albicans* as the most common pathogen causing invasive candidiasis [11, 12]. In Europe, the distribution of *Candida* species varied between countries [11]. The differences show that the incidence of invasive candidiasis may depend on the geographic region. The reasons for this are the differences in patient demographics (age, sex, etc) and the underlying diseases. In addition, the differences in medical practices such as diagnostic and therapeutic approaches, different antifungal treatment practices, use of invasive procedures and infection control approaches may contribute to the variation in fungal pathogen distribution. These factors have not been analyzed in this study.

Nevertheless, our study also separates the pathogen pattern based on etiological factor. In nosocomial infection with medical etiology patients, *C. albicans* (N = 17) has higher rate than *C. tropicalis* (N = 12). In nosocomial-surgical etiology, there was no difference in comparison (N = 2) for both *C. albicans* and *C. tropicalis*. Compare to patients with community infection, *C. tropicalis*has higher rate than *C. albicans*, both in medical (*C. albicans*, N = 14; *C. tropicalis*, N = 19) and surgical (*C. albicans*, N = 2; *C. tropicalis*, N = 6) ethiology. This shows that underlying diseases plays an important factor regarding the higher incidence of *Candida non-albicans* infection towards *Candida albicans*. This might be because the immune system of patients with community infections were already lower than patients with nosocomial infection since the first day of hospital admission, thus it tends to make them more vulnerable to *Candida non-albicans* infection. Further study needs to be conducted to confirm this finding. A higher incidence of gram-negative bacteria infections compared to gram-positive bacteria was found in our study. This is similar with a previous study conducted in 1999-2002 by Widodo et al [13]. The pattern of bacterial infection reported was similar with our study, with a difference in *Pseudomonas sp* and *Staphylococcus epidermidis* as the most common gram-negative and gram-positive bacteria pathogen, compared to *Klebsiella pneumoniae* and *Enterococcus faecalis* in our study. When we compared the pathogen pattern based on etiology, there were no difference between gram-negative bacteria pathogen found in nosocomial (medical and surgical) and community (medical and surgical) infection. The most common gram-negative bacteria found in both infections were *Klebsiella pneumoniae*. Another study by Radji M et al [14] also reported *P. aeurginosa*, *Klebsiella sp* and *E. coli* as the most common bacterial infection found in Fatmawati Hospital ICU, Jakarta. Study from Kariadi Hospital, Semarang, Indonesia also reported *P. aeruginosa*, *E. aerogenes* and *E. coli* as the most common bacterial infection [14]. It can be concluded that gram-negative bacterial infections are mainly responsible for nosocomial infections in Indonesian tertiary hospitals, despite slight variations in number. The differences might be caused by variations in the sample and underlying diseases.

## Conclusion

In conclusion, this study provided important epidemiological information on bacterial and fungal isolates in Indonesia's national tertiary hospital. There is a high incidence of *Candida tropicalis*, confirming the decreasing incidence of *Candida albicans* as dominant pathogen. Among critically ill patients with high risk for IFD, gram-negative bacteria were the most common bacteria isolated, with *Klebsiella pneumonia* being the most common bacteria. This shows that periodical revision of bacterial and fungal studies are needed to improve patient management and infection control. Data from our study gives important information for the right choice of empirical antibiotics and antifungals directed towards those pathogens in critically ill patients.

### What is known about this topic

- Bacterial infection and invasive fungal disease are major causes of morbidity and mortality in the critically ill patients;
- Gram-negative bacteria as the most common bacterial pathogen identified in critically ill patients.

### What this study adds

- The pattern of organisms causing infections varies between different countries and hospitals. This study provide data on bacterial and fungal pathogenic patterns in national tertiary care hospital;
- Candida tropicalis was found as the most common fungal pathogen in Indonesia tertiary care hospital, which confirmed the rise of Candida non-albicans infection in Asia region especially Indonesia;
- Data from this study can be used as a guidance for rationale early antibiotic and antifungal therapy.

## Competing interests

The authors declare no competing interests.

## Authors’ contributions

Gurmeet Singh contributes in study conception or design, data acquisition, data analysis or interpretation, manuscript drafting, critical manuscript revision and final manuscript approval. Stephanie Gita Wulansari contributes in study conception or design, data acquisition, data analysis or interpretation, manuscript drafting, critical manuscript revision and final manuscript approval. All the authors have read and agreed to the final manuscript.
